# Dynamics of cell cycle proteins involved in *Toxoplasma gondii*-induced bovine NET formation

**DOI:** 10.3389/fimmu.2023.1125667

**Published:** 2023-02-16

**Authors:** Zahady D. Velásquez, Raquel Peixoto, Ulrich Gärtner, Carlos Hermosilla, Anja Taubert, Iván Conejeros

**Affiliations:** ^1^ Institute of Parasitology, Biomedical Research Center Seltersberg (BFS), Justus Liebig University Giessen, Giessen, Germany; ^2^ Institute of Anatomy and Cell Biology, Justus Liebig University Giessen, Giessen, Germany

**Keywords:** NETs, cell cycle, PMN, cattle, bovine, Ki-67, lamin B1, centrosome

## Abstract

Neutrophil extracellular traps (NET) formation is one important host innate defense mechanism elicited by polymorphonuclear neutrophils (PMN). NETs are composed by chromatin and proteins with microbicidal and signaling activity. So far, there is one report on *Toxoplasma gondii-*triggered NETs in cattle, however, exact mechanisms, including signalling pathways and dynamics governing this reaction remain largely unknown. Recently, involvement of cell cycle proteins was demonstrated for phorbol myristate acetate (PMA)-triggered human PMN-derived NETs. Here, we studied the involvement of cell cycle proteins in *T. gondii*-induced NETs in exposed bovine PMN. Through confocal and transmission electron microscopy we discovered that Ki-67 and lamin B1 signals are upregulated and relocated during *T. gondii*-induced NETosis. Nuclear membrane disruption was also observed as a hallmark of NET formation in bovine PMN confronted with viable *T. gondii* tachyzoites, mimicking some steps of mitosis. However, we did not observe centrosome duplication as previously described for human PMN-derived NET formation stimulated with PMA.

## 1 Introduction

*Toxoplasma gondii* is a relevant zoonotic apicomplexan parasite of warm-blooded animals including humans. As such, at least one-third of the human population is currently infected with *T. gondii* worldwide (1). One of the most important sources for accidental human infections is the consumption of raw and/or undercooked meat derived from various livestock species carrying *T. gondii* cysts. Unlike sheep, goats and humans, natural *T. gondii*-infection in cattle does not appear to give rise to clinical signs or abortion in pregnant cows (1–3). However, *T. gondii* is also potentially pathogenic for cattle and known to induce anorexia, low milk production, myositis, neurological signs (i. e. depression), fever and mastitis (2). More recently, a quantitative microbial risk assessment (QMRA) model identified under-cooked/raw beef as the potentially most relevant source of *T. gondii* infections for the Dutch human population (4), thereby emphasizing the formerly disputed role of cattle in alimentary human Toxoplasma infections.

Considering the importance of cattle as source of infection, it seems relevant to understand early innate immune responses against *T. gondii* in the bovine system. Innate immunity is an early primary defence mechanism to prevent pathogens from systemic spread in the body (5). A major cellular component of the mammalian innate immune system are polymorphonuclear neutrophils (PMN). Defined as professional phagocytes, PMN display several mechanisms to kill and trap pathogens by releasing neutrophil extracellular traps (NETs), reactive oxygen species (ROS) production, phagocytosis, cytokine/chemokine production and degranulation (6). NET formation is an important and ancient effector mechanism of the innate immune system, elicited by activated PMN (7). NET structures are formed by chromatin (DNA) being decorated by enzymes with microbiocidal activities originating from PMN granules. The range of molecules and organisms that trigger NETs has increased immensely, however the exact molecular mechanisms behind NETosis are not entirely elucidated (8–10).

Firstly reported as a defence mechanism against bacteria and induced by soluble mediators, such as PMA and IL-8 (7), NET formation (NETosis), has meanwhile also been proven as an important defence mechanism against apicomplexan parasites (11, 12). Likewise, PMN of various mammalians, such as humans, sheep, cattle, dolphins, mice and seals confronted with *T. gondii* tachyzoites show enhanced production of ROS and/or NET formation (13–17). Interestingly, the latter immune reaction proved to block host cell invasion by *T. gondii* tachyzoites and thus inhibited parasite propagation (15). In cattle, *T. gondii* infections are often transient and host innate immune responses most probably lead to parasite elimination, however, the underlying molecular mechanisms are still poorly understood (18).

NET formation requires a sequence of events involving translocation of neutrophil elastase (NE) and myeloperoxidase (MPO) (19), chromatin decondensation, rupture of the nuclear membrane, fusion of cytoplasmic PMN vesicles with membranes, and finally, extrusion of NETs (20, 21). Some of these steps resemble a mitotic cell, notably chromatin decondensation and nuclear membrane rupture. Given that PMN are considered as terminally differentiated cells, it was recently hypothesized that this immune cell type repurposes cell cycle proteins to produce NETs. These proteins included Ki-67 as a proliferation marker, γ-tubulin as a centrosome marker and lamin B as a nuclear envelope integrity-related protein (22). So far, this hypothesis has neither been studied in parasite-triggered NETosis nor in the bovine system.

## 2 Materials and methods

### 2.1 Ethical statement

The experiments were conducted following the Justus Liebig University Giessen Animal Care Committee Guidelines. Protocols were approved by Ethics Commission for Experimental Animal Studies of Federal State of Hesse (Regierungspräsidium Giessen; A2/2016; JLU-No. 589_AZ and G16/2017, JLU-No. 835_GP) and in accordance to European Animal Welfare Legislation: ART13TFEU and current applicable German Animal Protection Laws.

### 2.2 *Toxoplasma gondii* tachyzoite maintenance

*Toxoplasma gondii* (RH strain) tachyzoites were maintained by serial passages in MARC-145 (Meat Animal Research Center-145) layers. The infection rate in MARC was 40-50% in all experiments. Therefore, free-released *T. gondii* tachyzoites were harvested from MARC supernatants, pelleted (400 × g, 12 min), counted in a Neubauer chamber, suspended in HBSS and used for bovine PMN confrontation. All experiments were performed at an MOI of 1:2 (bPMN:parasites).

### 2.3 PMN isolation

Healthy adult dairy cows (*n* = 4) served as blood donors. Animals were bled by puncture of the jugular vein and 30 ml blood were collected in heparinized sterile plastic tubes (Kabe Labortechnik). Twenty ml of heparinized blood were diluted in 20 ml sterile PBS with 0.02% EDTA (SigmaAldrich), layered on top of 12 ml Biocoll separating solution (density = 1.077 g/l; Biochrom AG) and centrifuged (800 × g, 45 min). After removal of plasma and PBMC, the cell pellet was suspended in 5 ml of Hank´s balanced salt solution (HBSS) and gently mixed with 1 volume of ice-cold phosphate-based lysis buffer (5.5 mM NaH_2_PO_4_, 8.4 mM HK_2_PO_4_, pH 7.2) for 1 min to lyse erythrocytes. Osmolarity was rapidly restored by adding 2 volumes of hypertonic buffer (5.5 mM NaH_2_PO_4_, 8.4 mM HK_2_PO_4_, 0.46 M NaCl, pH 7.2) and completing to 50 ml with HBSS. For full erythrocyte lysis, this step was repeated twice and PMN were washed twice in sterile HBSS. All centrifugation steps were performed using 600 × g for 6 min at 4 °C as previously described (23, 24). PMN were counted in a Neubauer haemocytometer. Finally, freshly isolated bovine PMN were allowed to rest (37 °C, 5% CO_2_ atmosphere) for 30 min before the experiments were performed (25).

### 2.4 Immunofluorescence microscopic analysis of cell cycle-related proteins in bovine PMN

Non-stimulated bovine PMN (negative control) and PMN confronted with *T. gondii* tachyzoites (1:2) were fixed at 15, 30 or 60 min of co-incubation depending on the experiment with paraformaldehyde 4% for 15 min at room temperature (RT). As negative control HBSS was used. As a positive control of NET formation, the calcium ionophore A23187 was used at a concentration of 25 µ M. The samples were carefully washed thrice with sterile PBS and incubated in blocking/permeabilization solution (PBS containing 3% BSA, 0.3% Triton X-100; Sigma-Aldrich) for 1 h at RT. Thereafter, the samples were incubated with the primary antibodies indicated in the **Table 1** diluted in blocking/permeabilization solution overnight at 4 °C in a humidified chamber. Thereafter, samples were washed thrice in sterile PBS (Sigma-Aldrich) and incubated in secondary antibody solutions (**Table 1**) for 30 min at RT, protected from light. Nuclear counterstaining was achieved by 4′,6-diamidin-2-phenylindol (DAPI; Sigma-Aldrich) present in mounting medium (Fluoromount G, ThermoFisher).

**Table 1:** Primary and secondary antibodies used for immunofluorescence analyses.

Primary Antibodies				
Antigen	Source	Cat #	Host	Dilution
Histone H3 S10	Abcam	ab5176	Rabbit	1:100
Ki-67	Abcam	ab15580	Rabbit	1:100
γ-tubulin	Abcam	ab179503	Rabbit	1:100
*Toxoplasma gondii*	ThermoFisher	PA1-7256	Goat	1:100
Histone-DNA	Millipore	MAB 3864	Mouse	1:200
Neutrophil elastase	Abcam	Ab68672	Rabbit	1:200

Secondary Antibodies				
Fluorophore	Company	Cat #	Reactivity	Dilution
AlexaFluor 488	ThermoFisher	A11008	Rabbit	1:500
AlexaFluor 594	ThermoFisher	A11005	Mouse	1:500
AlexaFluor 594	ThermoFisher	A11058	Goat	1:500

Images were acquired with a Zeiss Confocal LSM 710 equipped with a motorized XY stage and oil 63× objective (numerical aperture of 1.4) or in a Nikon Eclipse Ti2-A inverted microscope equipped with ReScan confocal microscopic instrumentation (RCM 1.1 Visible, Confocal.nl) and a motorized z-stage (DI1500). Three channels were recorded for signal detection: Blue/DAPI/405-laser, AlexaFluor488/Green/Argon-laser, and AlexaFluor594/Red/HeNe-543 laser. Images were acquired with a digital camera controlled by Zeiss ZEN 2010 software or using the NIS-Elements v 5.11 software (Nikon). Samples were imaged by z-stack optical series with a step-size of 0.3-0.5 microns depth. The z-series were displayed as maximum z-projections, and gamma, brightness, and contrast were adjusted (identically for compared image sets) using Image J software, FIJI version (26).

### 2.5 Image analysis and determination of the percentage of cells releasing NETs

Measurements of defined parameters (e.g. area, integrated density, number) were performed with FIJI/ImageJ software (version: 1.53c) (26). The percentage of cells releasing NETs was assessed as described by Brinkmann et al. (27) with minor modifications. Three random images per animal (*n* = 3) were taken at 10× magnification using a confocal microscope. Histone-DNA and DAPI signals were acquired at the same time for each image. A manual threshold was applied to each channel using the clustering algorithm of Otsu (28) and the total number of particles was counted. NETosis percentage was calculated using the following formula:


$$Cells\;releasing\;NETs\;\left(\%\right)=\frac{100\;\times N{}^{\circ}\;of\;cells\;in\;histone-DNA\;channel}{N{}^{\circ}of\;cells\;in\;DAPI\;channel}$$

To determine the fluorescence intensity of cell cycle proteins used in this study, z-stack images of each channel were projected to obtain a single image using the maximum projection algorithm. The segmentation workflow consisted in two steps. First the cell boundaries were defined in the DIC channel to select the region of interest (ROI) which belongs to each cell using the following parameters: Gaussian blur 2 pixels, subtract background radius 30 pixels, threshold Otsu, fill holes binary process, analyzed particles over 20 pixels and circularity 0.5-1. Then, the fluorescence intensity in the green channel of the pre-defined ROI was determined as raw integrated density.

A schematic workflow of the image analysis is shown in the **Supplementary Figure 1**.

### 2.6 Transmission electron microscopy

For TEM analysis, 1 x 10^7^ bovine PMN were confronted with vital 2 x 10^7^ *T. gondii* tachyzoites, and after 2 h of co-incubation, cells were fixed in glutaraldehyde (2.5% final concentration, Merck). The cells were centrifuged at 1000 × *g* for 10 min and the pellet was stored at 4 °C until further use according to (7) and (29). Briefly, the cell pellet was washed and post-fixed in buffer containing 1% osmium tetroxide (Merck). After thoroughly washing in distilled water, the samples were incubated overnight in 2% aqueous uranyl acetate (Merck) at 4 °C, dehydrated in ethanol and embedded in Epon (all Merck). Ultrathin sections of the cured blocks were mounted on formvar-coated grids and stained with uranyl acetate and Reynolds lead citrate (both Merck). The sections were inspected in a ZEISS EM912 AB microscope (Oberkochem, Germany) at the Institute of Anatomy and Cell Biology of the Justus Liebig University Giessen, Germany.

### 2.7 Statistical analyses

Hypothesis testing was performed by a Mann-Whitney test with a confidence level of 95%. All graphs (mean ± SD) and statistical analyses were performed in the Graph Pad software (v. 7.03).

## 3 Results

### 3.1 *T. gondii* tachyzoites induce the release of bovine NETs

**Figure 1:**
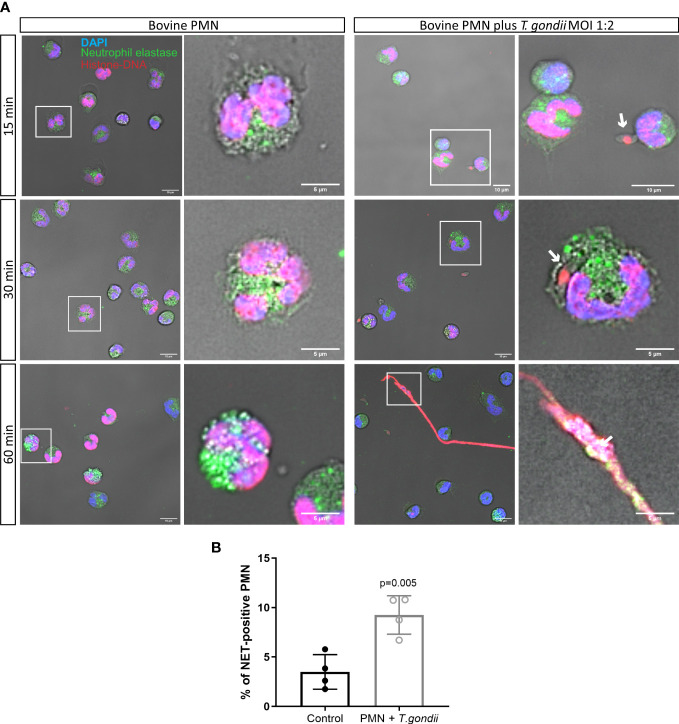
*T. gondii*-induced NET release in bovine PMN. 2 × 10^5^ bovine PMN were confronted with vital 4 × 10^5^ *T. gondii* tachyzoites (1:2 ratio) for 60 min. Samples were fixed in PFA 4% after 15, 30 and 60 min of co-culture and the specimens were stained for neutrophil elastase (NE; green channel), histone-DNA complex (red channel) and DNA (blue channel) **(A)**. The percentage of cells releasing NETs was determined at 60 min of parasite confrontation as described in Material and Methods section and represented as bar graph (mean ± SD). White squares indicate the region of the image zoomed in the corresponding right panel. White arrows indicate *T. gondii* tachyzoites in direct contact with PMN or trapped on NETs. **(B)**. *P*-value was estimated by a Mann Whitney test (α = 0.05).

NET formation of bovine PMN being confronted with *T. gondii* tachyzoites was studied at 15, 30 and 60 min of interaction. The percentage of NET-positive cells was determined using an automated protocol and using NE- and DNA-related fluorescence as reference to define NET structures according to Brinkmann et al. (27). Therefore, immunofluorescence using anti-Histone-DNA and anti-NE antibodies was used (**Figure 1A**). After 60 min of interaction, NET structures showed co-localization of both parameters and resulted in typical NET-related characteristics, such as membrane-unbound and spread chromatin (**Figure 1A**, 60 min). Quantification of NET-positive cells unveiled 9.25% of bovine PMN undergoing a NETotic process after 60 min of exposure to *T. gondii* tachyzoites in a 1:2 ratio (**Figure 1B**). In non-stimulated bovine PMN controls, 4% of NETs-positive cells were found using this quantification method. Stimulation of PMN with the calcium ionophore A23187 was used as positive control, inducing 33% of NET-positive cells after 60 min (**Supplementary Figure 2**).

### 3.2 *T. gondii* tachyzoite exposure induces Ki-67 upregulation in bovine PMN

**Figure 2:**
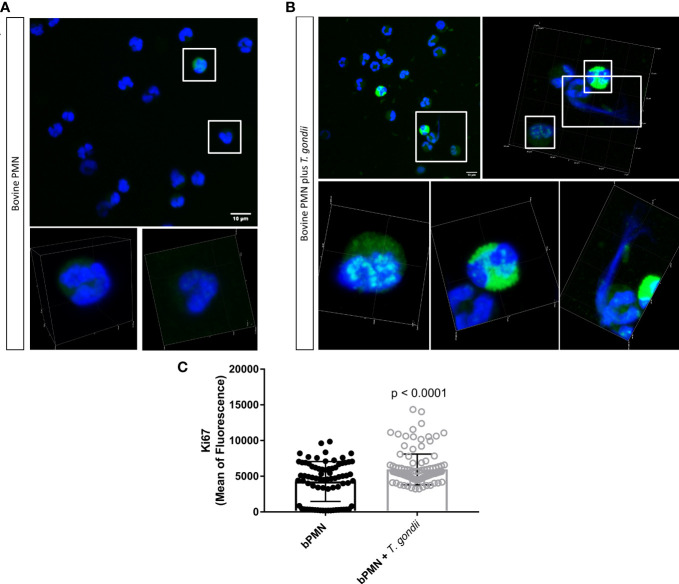
Abundance of the mitotic marker Ki-67 in *T. gondii* tachyzoite-exposed bovine PMN. 2 × 10^5^ bovine PMN were confronted with vital 4 × 10^5^ *T. gondii* tachyzoites (1:2 ratio) for 60 min. Samples were fixed in PFA 4% at 60 min of interaction and the specimens were stained for Ki-67 (green channel) and DNA (blue channel; DAPI). White squares illustrate zoomed cells in the corresponding lower panel **(A, B)**. The abundance of Ki-67 was determined based on fluorescence signals detected via confocal microscopy and represented as bar graph (mean ± SD). Differences in Ki-67 abundance between unstimulated and *T. gondii*-confronted PMN **(C)** were analysed by a Mann Whitney test (α = 0.05).

Signals of the proliferation marker Ki-67 in tachyzoite-exposed bovine PMN were monitored at 60 min of interaction by confocal microscopy. Non-stimulated cells were used as negative controls. After parasite exposure, an upregulation in abundance of Ki-67-derived fluorescent signals was observed. Unstimulated PMN presented either low Ki-67 related fluorescence signals (always associated with the PMN nuclei; **Figure 2A**, zoomed cells in white squares and **Supplementary Video 1**) or non at all. On the other hand, parasite-exposed PMN showed upregulation of Ki-67 (*p* ≤ 0.001) after 60 min of interaction (**Figure 2B**, zoomed cells in white squares and **Supplementary Video 2**) thereby being correlated with the NET release-related time point.

### 3.3 NET formation occurs without centrosome duplication in *T. gondii*-confronted bovine PMN

**Figure 3:**
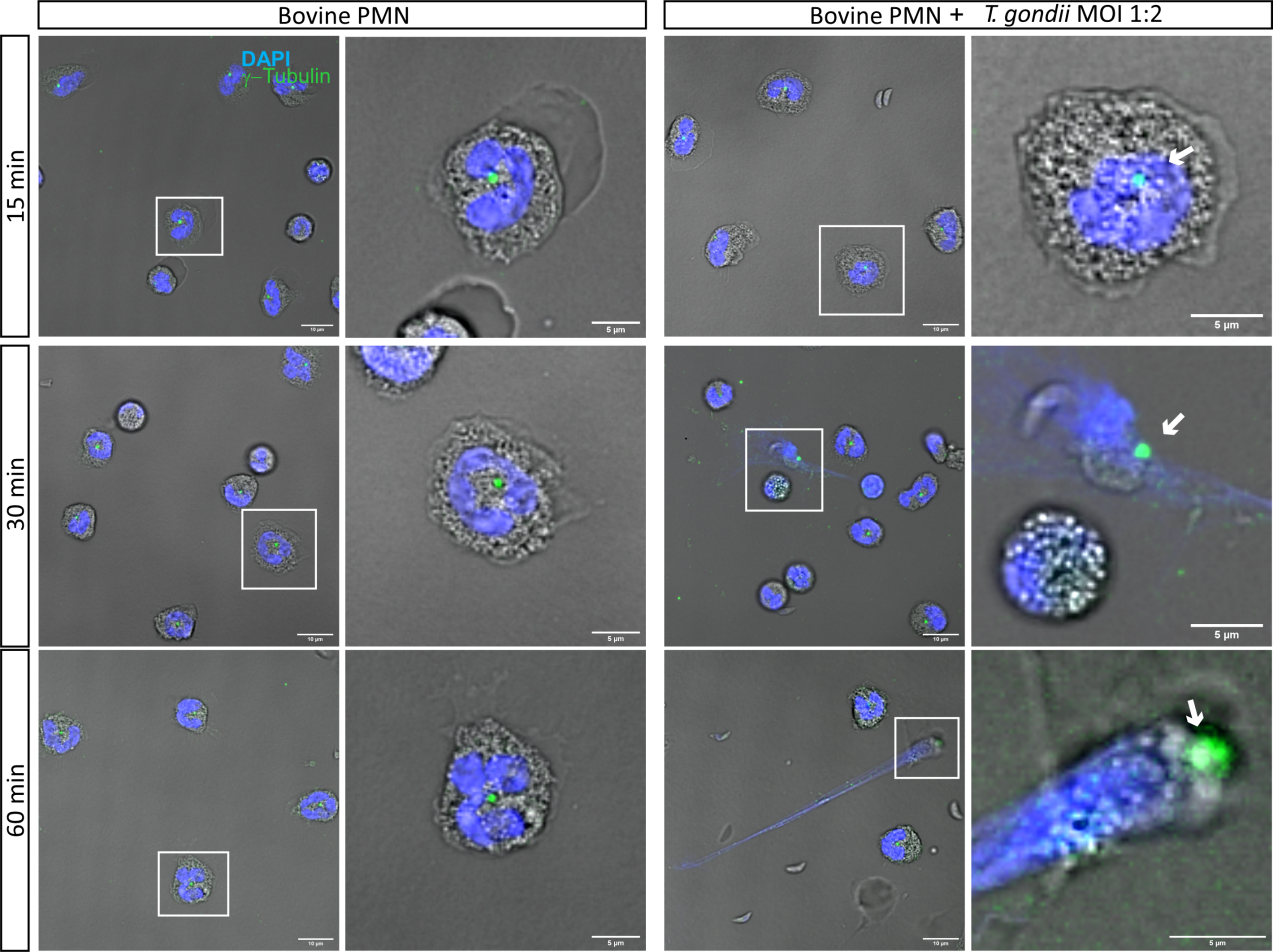
*T. gondii* tachyzoite exposure does not induce centrosome duplication in bovine PMN. 2 × 10^5^ bovine PMN were confronted with vital 4 × 10^5^ *T.gondii* tachyzoites (1:2 ratio) for 60 min. Samples were fixed in PFA 4% at 15, 30 and 60 min of co-culture and the specimens were stained the centrosome marker γ-tubulin (green channel) and DNA (blue channel; DAPI). In white squares the cells which are zoomed in the corresponding right panel are shown. Centrosomes are indicated by a white arrow. Scale bars correspond to 10 µm and 5 µm in zoomed images.

Given that it was previously described that PMA-stimulated PMN duplicate centrosomes when forming NETs (22), we used the centrosome marker γ-tubulin to follow centrosomal structuring during activation of *T. gondii*-confronted PMN at 15, 30, 60 and 120 min of co-incubation (**Figure 3**, **Supplementary Figure 3**). The centrosome is visualized as a green dot in very close proximity to nuclear chromatin (stained with DAPI-mediated blue fluorescence). Current data show that centrosome duplication is absent in *T. gondii*-confronted bovine PMN (**Figure 3**). Likewise, this finding was also observed when the co-incubation time was extended to 120 min. (**Supplementary Figure 3**).

### 3.4 Phosphorylated Histone H3-Serine 10 (p-HH3S10) is upregulated in *T. gondii* tachyzoite-exposed bovine PMN

**Figure 4:**
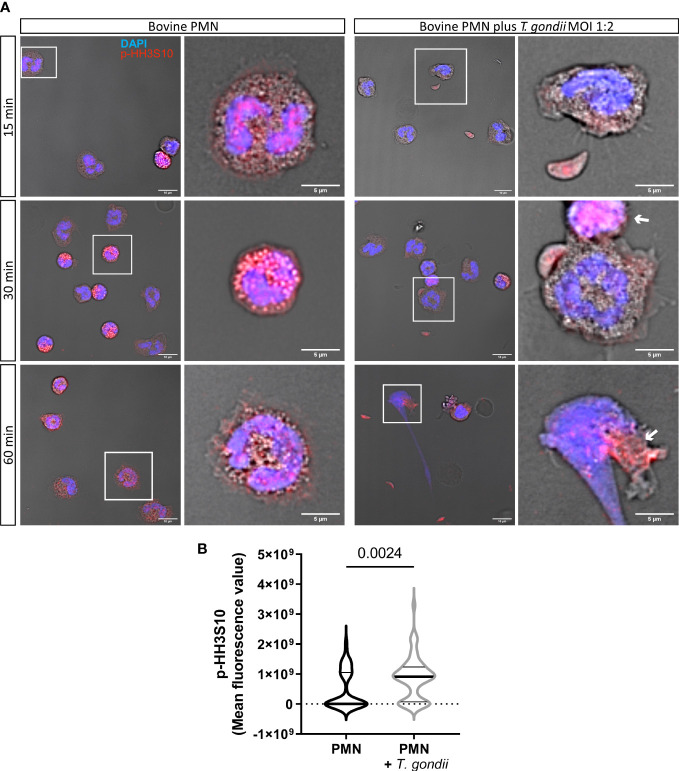
*T. gondii* induces upregulation of the mitotic marker p-HH3S10 in exposed bovine PMN. 2 × 10^5^ bovine PMN were confronted with vital 4 × 10^5^ *T. gondii* tachyzoites (1:2 ratio) for 60 min. Samples were fixed in PFA 4% at 15, 30 and 60 min of interaction and the specimens were stained for the mitotic marker p-HH3S10 (red channel) and DNA (blue channel; DAPI). In white squares the cells which are zoomed in the corresponding right panel are shown **(A)**. The abundance of p-HH3S10 was determined at 60 min based on fluorescence signals detected by confocal microscopy and represented as violin plot (line at mean). Differences in the expression of p-HH3S10 at 60 min of interaction were analysed by a Mann-Whitney test (α = 0.05) **(B)**. White arrows indicate PMN with higher and relocated abundance of p-HH3S10.

Next, we evaluated the abundance of the mitotic marker p-HH3S10 in NETotic PMN after 15, 30 and 60 min of co-incubation with *T. gondii* tachyzoites (**Figure 4A**). Here, the phosphorylated form of HH3 in bovine PMN was detected as early as 30 min after tachyzoite exposure. Quantification of p-HH3S10-derived fluorescent signals evidenced an increased neutrophil abundance of p-HH3S10 (*p* = 0.0024) at 60 min of *T. gondii* exposure (**Figure 4B**), thereby indicating that the mitotic machinery was indeed active in NETotic bovine PMN.

### 3.5 Lamin B1 localization in *T. gondii*-confronted PMN

**Figure 5:**
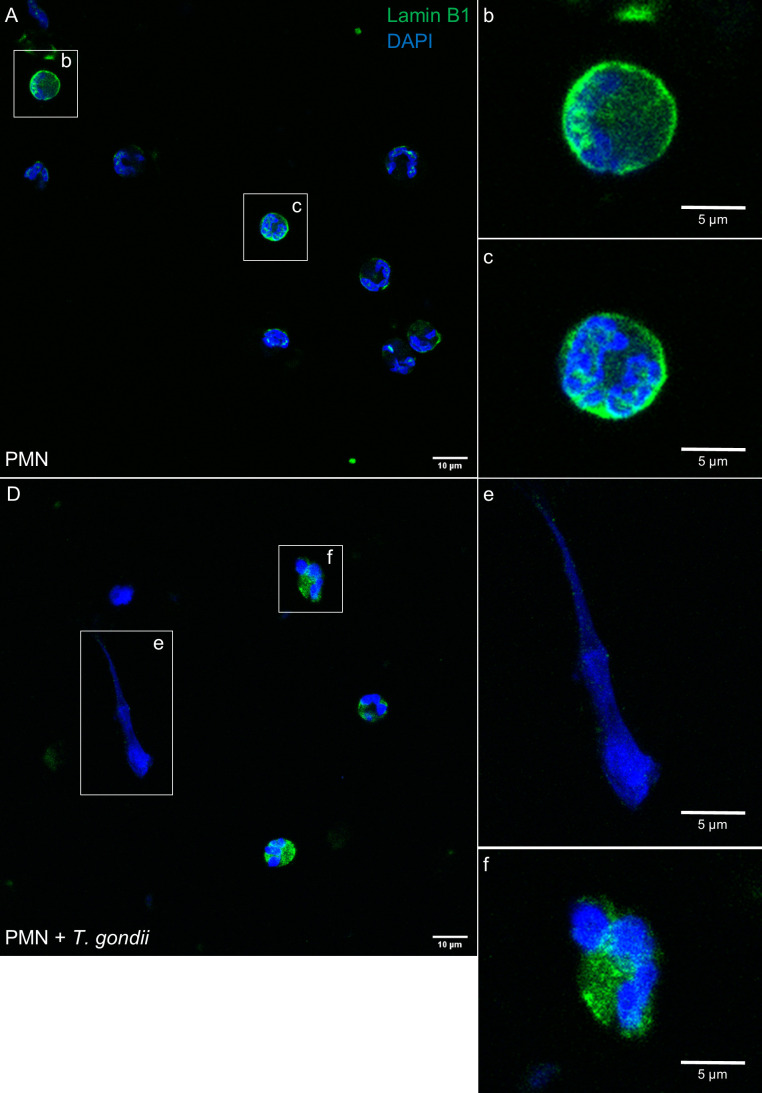
*T. gondii* induces relocation of lamin B1 in bovine stimulated PMN. 2 × 10^5^ bovine PMN were confronted with vital 4 × 10^5^ *T. gondii* tachyzoites (1:2 ratio) for 60 min. Samples were fixed in PFA 4% and the specimens were stained for lamin B1 (green channel) and DNA (blue channel; DAPI). Unstimulated PMN **(A)** shows a perinuclear distribution of lamin B1 **(B, C)**. *T. gondii*-confronted PMN **(D)** show in contrast, a loss of continuous perinuclear lamin B1 signals when forming NETs **(E)** or relocation when the chromatin is decondensed **(F)**. Images are representative of 3 (n=3) individual experiments.

The abundance and distribution of the nuclear lamina marker lamin B1 was also studied at 60 min after PMN exposure to *T. gondii* tachyzoites. In control PMN, lamin B1 signal showed a homogeneous perinuclear pattern indicating intact nuclear membranes (**Figure 5A–C**). In contrast, when the PMN are confronted to *T. gondii* tachyzoites (**Figure 5D**), changes in lamin B1-derived fluorescence signals indicate nuclear membrane disruption since the signal are relocated (**Figure 5F**) or even lost when the PMN are forming NETs (**Figure 5E**).

### 3.6 Transmission electron microscopy analysis illustrates typical features of NETs accompanied by disruption of the nuclear membrane in *T. gondii*-exposed PMN

**Figure 6:**
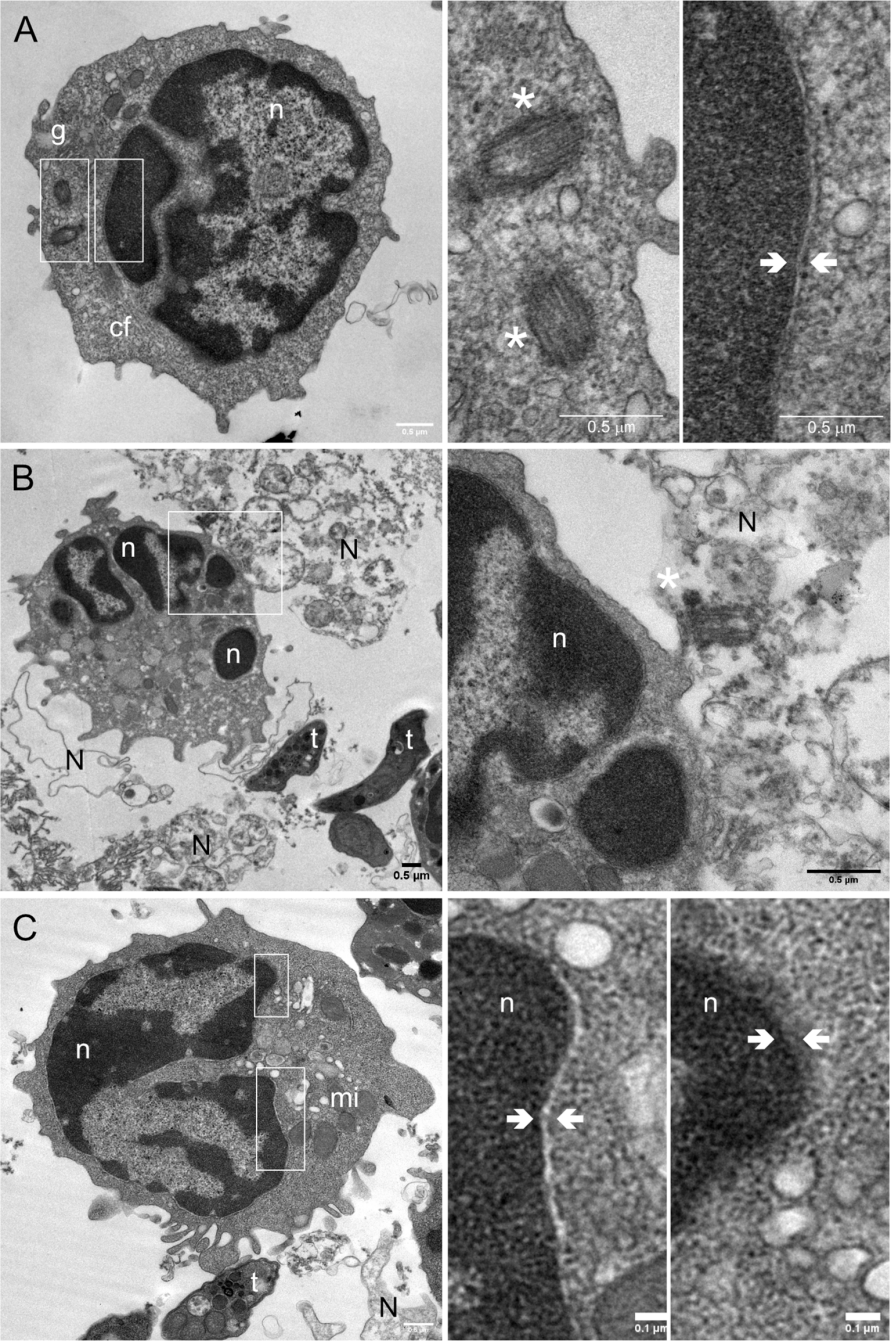
*T. gondii* tachyzoite-induced nuclear membrane disruption in exposed bovine PMN illustrated by transmission electron microscopy (TEM). 2 × 10^5^ bovine PMN were confronted with vital 4 × 10^5^ *T. gondii* tachyzoites (1:2 ratio) for 60 min. Samples were fixed in glutaraldehyde 2.5% at 60 min of incubation and analysed by TEM. Non-stimulated PMN (= controls) **(A)** show typical ultrastructural details of PMN, such as a multilobulated nucleus (n), distinct subtypes of granules (g) and cytoskeletal filaments (cf). White asterisks indicate the centrosomes, and the white arrows highlights the nuclear membrane. **(B)** After 60 min of exposure to *T. gondii* tachyzoites (t), bovine PMN released NETs (N) and centrosomes were observed associated with the DNA (*). NET formation was accompanied by disruption of the nuclear membrane **(C)**; mi = mitochondria. White boxes indicate the regions of the image that are zoomed out in the corresponding right panels.

To verify confocal microscopy-related observations and to obtain further ultrastructural details of *T. gondii*-induced NET formation in bovine PMN, we also performed TEM analysis (**Figure 6**). The cell structures of interest are indicated in non-stimulated bovine PMN as follows, g: granules, cf: cytoskeleton filaments, n: nucleus, white asterisk (*): centrosome and nuclear membrane indicated by white arrows (**Figure 6A**). *T. gondii*-confronted PMN (**Figure 6B**) showed NETs (N) being extruded from PMN to the extracellular space where *T. gondii* tachyzoites (t) were present. Interestingly, the centrosome (asterisks) was present in NET structures in close proximity to the PMN (white square; zoomed in the right panel). **Figure 6C** illustrates a discontinuous PMN nuclear membrane (white arrows) in two different nuclear regions of a NETotic PMN (white rectangles).

## 4 Discussion

The wide distribution of *T. gondii* in terrestrial and marine mammals documents the capability of this parasite species to successfully overcome the first line of defence in mammals, mainly represented by circulating PMN in the blood system and tissues. The main function of PMN, as part of the innate immune system, is to avoid the spread of invasive pathogens by several different killing and trapping mechanisms. In this context, NETs play an important role in trapping and killing not only bacteria (7, 30) and fungi (31, 32), but also apicomplexan parasites as already shown for *T. gondii*, *B. besnoiti* and *Neospora caninum* in the bovine system (13, 25, 33–35) and indicating that NET formation indeed is a conserved and ancient defence mechanism (36).

Information on cellular and molecular mechanisms behind NETosis as well as molecules involved in this defence process have increased steadily in the last decade (8). However, a vast majority of specific features and signalling pathways involved in parasite- and especially *T. gondii*-induced NET formation is still unknown. A previous report demonstrated for the first time an involvement of cell cycle-related proteins in NET formation induced by PMA and *Candida albicans* (22). Considering that PMN are terminally differentiated leukocytes, and, consequently, do not perform cell division and proliferation (22, 37), we here analyzed whether selected cell cycle proteins might be repurposed as a conserved feature in parasite (*T. gondii*)-induced suicidal NETosis, and, more importantly as a mechanism conserved among mammalian species (e. g. bovines and humans).

As already documented for several other host systems (13–15, 33, 35), *T. gondii* tachyzoites are able to induce NETosis in bovine PMN after 60 min of incubation. The proportion of PMN forming NETs (9.25%) is in line with similar values observed in case of *B. besnoiti* tachyzoites (15%) or bradyzoites (11%) (25, 38, 39). In the bovine system, higher levels of NETotic PMN were typically observed when soluble stimulants were used. For example, in case of the calcium ionophore A23187, almost 60% of PMN became NET-positive (38). Interestingly, host species-specific differences in the kinetic and magnitude of NET reactions were reported for PMA (a molecule widely used as potent NET-inducer in literature) since bovine PMN reacted less and slower *via* NETosis towards this molecule than human PMN (20, 35). The marked difference in the magnitude of general PMN-related immune responses between human and bovine PMN was already described decades ago (40), indicating that interpretation of NET-related data must take always into account the PMN donor species, incubation time and nature of the stimulus. We here chose to study NET formation after 60 min of exposure in order to focus on early events of cell cycle protein repurposing in *T. gondii*-induced NETosis, and to avoid secondary activation events eventually leading to amplification of NET formation in PMN (41). Since we wanted to focus on the expression and localization of certain cell cycle proteins in parasite-activated PMN, we intentionally kept the PMN:*T. gondii* tachyzoites ratio relatively low (1:2) compared to previous reports (13, 14, 25, 42) in order to facilitate visualization of cell-to-cell contacts and imaging of rapid NETosis-related kinetics.

Overall, the abundance of the mitotic markers Ki-67 and pHH3S10 was monitored in *T. gondii-*stimulated PMN at 60 min. The increased Ki-67 expression after 60 min of tachyzoite exposure indicates an activation of mitotic events in PMN during *T. gondii-*induced NETosis. This result is concordant with the previous report on human PMN being stimulated with PMA (22). Notably, Ki-67-positive PMN were found in histological sections from patients with brain fungal abscesses (22), confirming the importance of this cell cycle marker at the initial phase of NETosis *in vivo*. Bovine NETosis was also accompanied by an increased abundance of pHH3S10, which is also in line with findings on human PMN stimulated with PMA (22). Thus, at least these two proteins are involved in early *T. gondii*-induced NETosis in bovine PMN.

In the current work, centrosome duplication was also studied in *T. gondii*-induced NET formation using the specific marker γ-tubulin. However, in contrast to findings on PMA-triggered human NETosis (22), no centrosome duplication was detected, neither in PMA-stimulated nor in *T. gondii*-confronted bovine PMN. Intriguingly, in the work of Amulic et al. (22) centrosome duplication was also detected *in vivo* in histological sections of patients suffering from fungal brain abscesses thereby reflecting totally different experimental scenarios. It is unclear why centrosome duplication does not occur in *T. gondii* exposed PMN.

Nuclear envelope breakdown is a hallmark of suicidal NETosis allowing for the differentiation of other cell death mechanisms, such as necrosis or apoptosis (22). Current data showed that exposure of bovine PMN to vital *T. gondii* tachyzoites for 60 min induced a relocation or loss of the nuclear membrane marker lamin B1 when PMN showed decondensed chromatin or were forming NETs. Furthermore, a discontinuity of the PMN nuclear membrane was clearly illustrated in *T. gondii*-exposed bovine PMN *via* TEM analysis.

**Figure 7:**
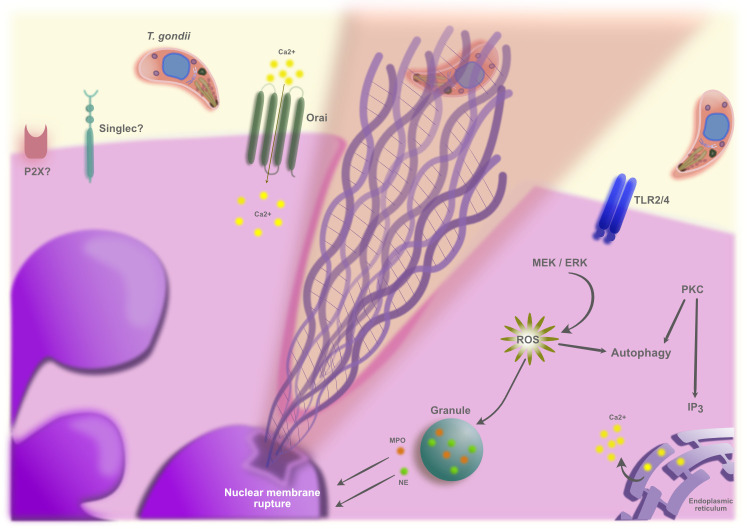
Schematic representation of the possible PMN signalling pathways involved in *T. gondii*-induced NET formation.

Regarding possible signalling pathways involved in *T. gondii*-induced NETs, to date evidence exists showing the involvement of TLR2/4 (43, 44), MAPK, ROS, store operated calcium entry (SOCE) as well NE and MPO (35, 42, 45). It is also possible to speculate that other signalling pathways observed to play a role in apicomplexan parasites-induced NETs as purinergic signalling (34, 46) and carbohydrate motifs recognition by PMN lectins are also playing a role in *T. gondii*-induced NETs. A scheme summarizing these signalling pathways is shown in the **Figure 7**.

In summary, we here confirmed *T. gondii*-induced bovine NET extrusion and showed the involvement of selected cell cycle-related proteins. The early events (< 60 min post-confrontation) of PMN undergoing NETosis included disruption of the nuclear membrane previous to the chromatin release into extracellular space. This report adds novel data to the expanding field of apicomplexa-induced NETosis, specifically of the zoonotic-relevant *T. gondii*, and calls for more investigations on molecular signalling pathways involved in mammalian NET formation.

## Data availability statement

The raw data supporting the conclusions of this article will be made available by the authors, without undue reservation.

## Ethics statement

The animal study was reviewed and approved by Justus Liebig University Giessen Animal Care Committee Guidelines and the Ethics Commission for Experimental Animal Studies of Federal State of Hesse (Regierungspräsidium Giessen; A2/2016; JLU-No. 589_AZ and G16/2017, JLU-No. 835_GP).

## Author contributions

Conceptualization: IC, ZV and CH. Methodology: ZV. PMN isolation: RP. Validation: IC. Formal analysis: ZV. Investigation: ZV, UG. Resources: AT. Data curation: ZV, IC. Writing and original draft preparation: IC. Writing, review and editing: IC, CH, AT, ZV. Visualization: ZV, IC. Supervision: IC, AT. Funding acquisition: CH, AT. All authors contributed to the article and approved the submitted version.
