# Supplementary material for: Dynamics of cell cycle proteins involved in Toxoplasma gondii-induced bovine NET formation
Source: Front Immunol. 2023 Feb 16;14:1125667. doi: 10.3389/fimmu.2023.1125667 (PMC9981159; doi:10.3389/fimmu.2023.1125667)
Supplement: Supplementary file 1 [file DataSheet_1.docx]

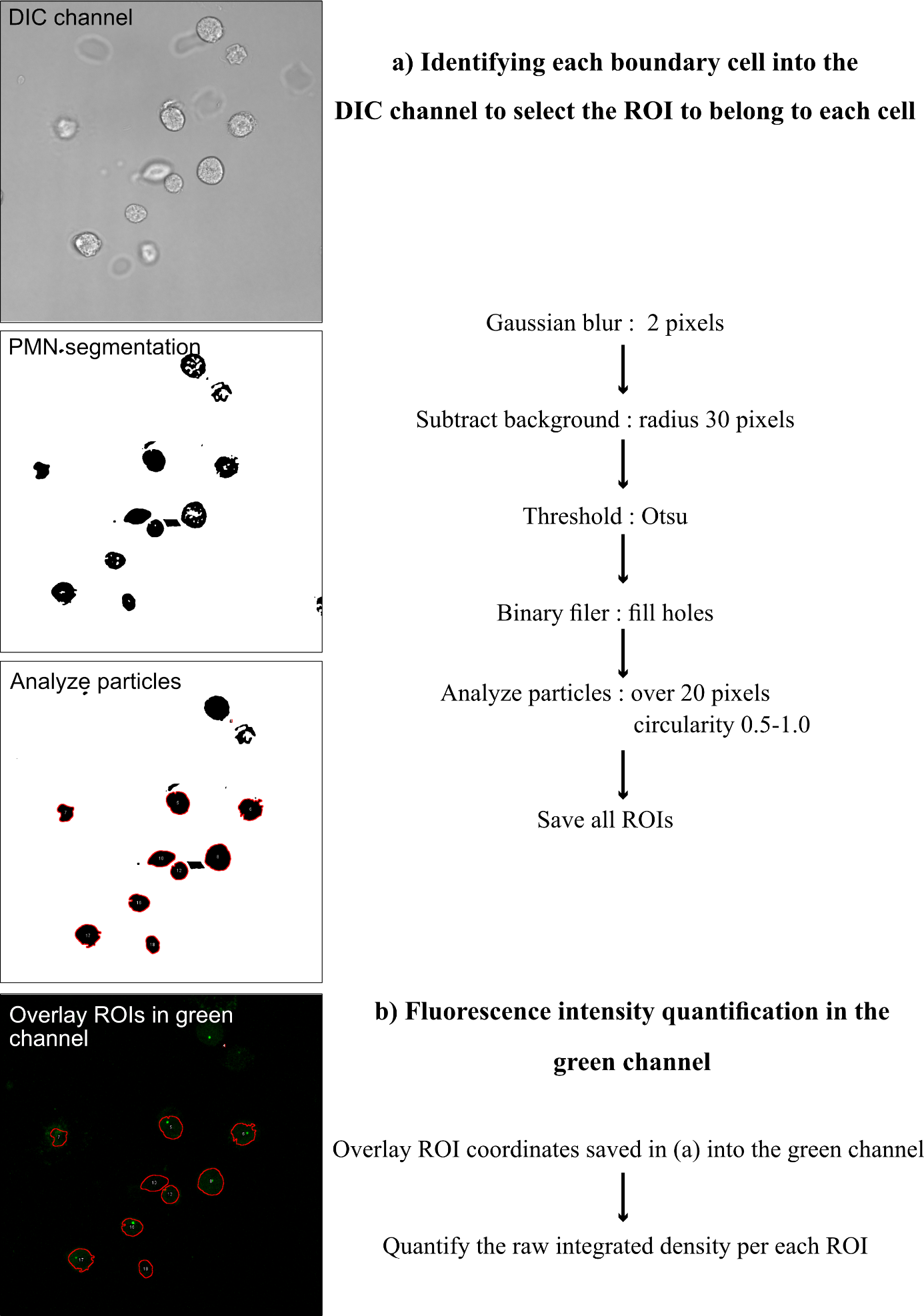


**Supplementary Figure 1.** Schematic overview of the workflow of the image analyses used to quantify the percentage of NETs as described in Material and Methods.


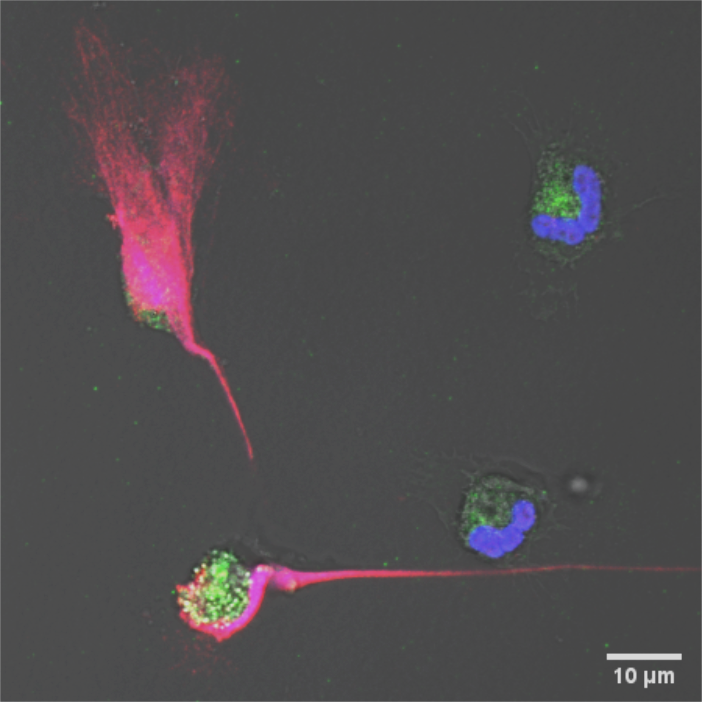


**Supplementary Figure 2. A23187 induces NETs in bovine PMN.** As positive control A23187 25 µM was used to stimulate 2 x 10^5^ bovine PMN. After 1h of incubation the samples were fixed in 4% PFA and immunostaining for NETs components was performed as described in Material and Methods.


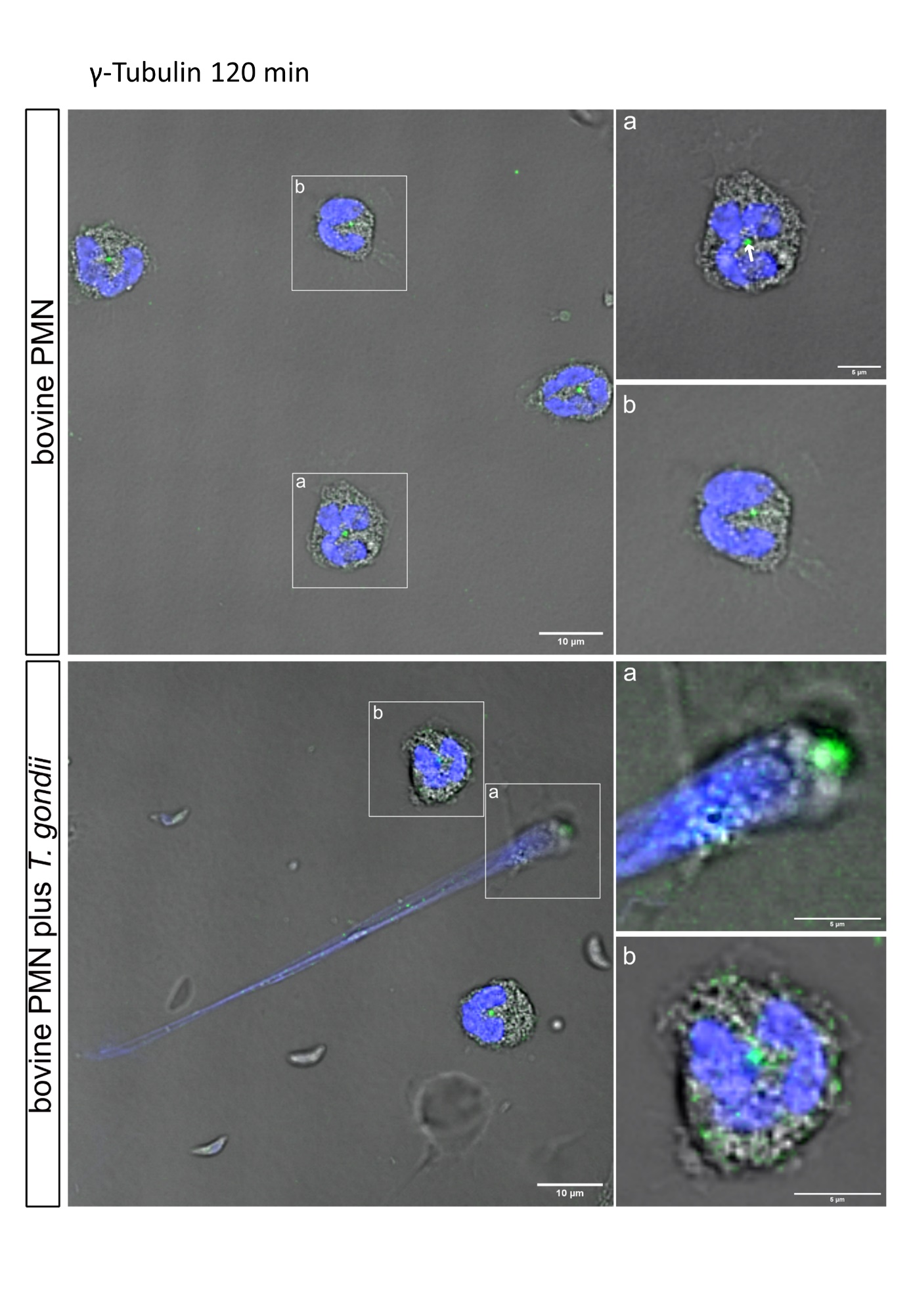


**Supplementary Figure 3.** γ-tubulin immunodetection on bovine PMN after 120 min of co-incubation with *T. gondii* tachyzoites. Blue: DAPI/DNA. Green: γ-tubulin
